# Simultaneous determination of 101 volatile organic compounds released from plastic runway tracks based on the environmental chamber-canister sampling-three-stage cold trap preconcentration-gas chromatography-mass spectrometry/flame ionization detection method

**DOI:** 10.3389/fchem.2025.1605810

**Published:** 2025-07-21

**Authors:** Gan Liu, Yong Ma, Hong Wang, Yanrong Meng, Yu Huang, Weitao Zheng

**Affiliations:** ^1^ Engineering Research Center of Sports Health Intelligent Equipment of Hubei Province, Wuhan Sports University, Wuhan, China; ^2^ Key Laboratory of Sports Engineering of General Administration of Sport of China, Wuhan Sports University, Wuhan, China

**Keywords:** plastic runway tracks, environmental chamber, gas chromatography, mass spectrometry, volatile organic compounds

## Abstract

This paper provides a strategy for detecting and monitoring volatile organic compounds released from plastic runway tracks. The method applies a simultaneous determination of 101 VOCs based on the environmental chamber-canister sampling-three-stage cold trap preconcentration-gas chromatography-mass spectrometry/flame ionization detection (GC-MS/FID) method. For this purpose, an environmental chamber, SUMMA canisters, an atmospheric pre-concentrator, and a GC-MS/FID dual detection setup were adopted to collect VOCs released from plastic athletic tracks in an environmental chamber, followed by their preconcentration in a three-stage cold trap including glass-bead cold trap concentration, Tenax tube cold trap concentration, and capillary glass tube absorption focusing. Qualitative and quantitative analyses of VOCs were conducted. The obtained results showed that the optimal environmental parameters for releasing VOCs from plastic runway tracks were an ambient temperature of 60°C, a relative humidity of 5%, an air exchange rate of 1.0 h^−1^, and a release time of 24 h. The established method showed a linear relationship within a range from 0.8 to 16.0 ppb, with linear correlation coefficients for different compounds ranging from 0.9546 to 1.0000. The detection limits of the method ranged from 0.01 to 0.74 μg·m^−3^ (equivalent to 0.005–0.220 ppb, at 60°C and 1 atm), the relative error (n = 7) was between −10.16% and 12.84%, and the relative standard deviation (n = 7) was from 0.16% to 4.94%. The released VOCs can be divided into seven categories, including alkanes, alkenes, alkynes, aromatic hydrocarbons, halogenated hydrocarbons, oxygenated organic compounds, and nitrogenous organic compounds. Acetone (Z)-1,2-dichloroethene, 3-methylheptane, n-octane, n-decane, n-butane, trans-2-pentene, styrene, and 1,1,2,2-tetrachloroethane were common VOCs contained in athletic plastic tracks. The established simultaneous determination of VOCs based on the environmental chamber-canister sampling-three-stage cold trap preconcentration-GC-MS/FID method showed good linear and correlation relationships, high sensitivity and precision, and strong repeatability, which is suitable for the qualitative and quantitative detection of 101 kinds of VOCs from plastic athletic tracks. Finally, it was concluded that small differences in the mass concentration of the main VOC monomers appear in different athletic plastic tracks.

## 1 Introduction

Plastic runway tracks are mainly composed of polyurethane (PU) adhesive, PU rubber particles, ethylene propylene diene monomer (EPDM) rubber particles, diluents, additives, organic hydrocarbons, and their derivatives. The diversity of raw materials facilitates the release of volatile organic compounds (VOCs) during their use for sports activities, and the VOCs may enter the human respiratory system through breathing; moreover, human bodies are also extensively exposed to these substances through skin pores (Wang et al., 2017; Liu et al., 2018a; Liu et al., 2018b; Liu et al., 2025). All this may cause teratogenic and carcinogenic effects, yielding severe diseases of the nervous and cardiovascular systems, liver, kidneys, and lungs, affecting the athletic ability of sportspersons (Liu et al., 2008; Cakmak et al., 2011; Kargarfard et al., 2015; Wcisło et al., 2016; Si and Cardinal, 2017; Sun et al., 2019).

Yang et al. (2018) and Li S. et al. (2021) used gas chromatography (GC) to determine the release rate of total volatile organic compounds (TVOCs) from plastic runway tracks and 9 VOCs from PU adhesive. However, Yang et al. (2018) demonstrated that thermal desorption in GC analysis can introduce significant errors: low-temperature desorption may result in incomplete desorption of TVOCs, while high temperatures can cause the adsorbent in the adsorption tube to degrade, compromising adsorption efficiency. Additionally, Li (2023) and Schanzmann et al. (2025) observed in actual GC-based detection processes that when the retention times of other components in VOC samples closely match those of the target compounds, multiple components may co-elute (particularly isomers or compounds with similar properties), leading to peak overlap and potential misidentification as the target compound, thereby producing false positives. Therefore, gas chromatography-mass spectrometry (GC-MS) was widely adopted for the qualitative and quantitative analysis of VOCs released from plastic runway tracks, including aldehydes, alcohols, ketones, alkanes, halogenated hydrocarbons, monoaromatic hydrocarbons, and many other compounds (Chang et al., 1999; Hu et al., 2017; Yuan et al., 2019; Kamarulzaman et al., 2019; Jiang et al., 2019; Cui et al., 2019; Guo et al., 2020; Li et al., 2020; Zhou et al., 2020). However, due to the variety of raw materials used to prepare plastic runway tracks and complex and changeable outdoor service environments, the qualitative and quantitative determination of VOCs is still incomplete, and many unknown or trace VOCs may appear (Li M. et al., 2017; Li Q. et al., 2021).

The sampling of VOCs released from plastic runway tracks is usually conducted using the methods of absorption in organic solvents or adsorption on specific adsorbents, but both methods require different absorbents and adsorbent fillers for different compounds, limiting the simultaneous determination of multiple components. In such situations, SUMMA canister sampling is favorable due to its ability to capture all components and their environmental friendliness. Li H. et al. (2017) used 6L and 15L SUMMA canisters for ambient air collection and cryo-trapping-GC-MS/FID (flame ionization detection, FID) for the automatic online monitoring of multi-component VOCs, showing that the method can quantify 89 VOCs from the ambient air, including 31 halogenated hydrocarbons, 45 hydrocarbons, and 13 oxygenated compounds. The linear correlation coefficients, *R* ^2^, for 89 VOCs were >0.95, the repeatability relative standard deviation (RSD) was ≤28% (RSD ≤15% for 83 VOCs), and the relative error (RE) was ≤30% (RE ≤ 15% for 76 VOCs). Xu (2017) established a method for determining 108 VOCs from ambient air by SUMMA canister sampling and a preconcentration system-combined GC/MS, showing good linearity in a range of 0.50–12.5 nmol·mol^−1^. The minimum detection limit was <0.29 nmol·mol^−1^, and the RSD was ≤9.2%. The method used only GC/MS for the detection and analysis of VOCs, which is able to carry out preliminary monitoring of the component characteristics of VOCs and meets the requirements of ambient air VOCs monitoring. Zhou and Feng (2018) used a canister sampling-GC-MS system to analyze 104 VOCs from the ambient air and optimized the ramp-up procedure and the three-stage cold trap temperature. The RSDs of the optimized method for all 104 VOCs were <10%, the method detection limits (MDL) ranged from 0.02 to 0.26 nmol·mol^−1^, the retention time RSD was <0.05%, and the concentration RSD was <7%. Liang et al. (2021) used SUMMA canister sampling-air preconcentration, a gas phase cold oven, and a GC-MS system to achieve the simultaneous determination of 57 PAMS and 65 TO-15. The detection ranges for 108 VOCs were linear, between 0.15 and 8.0 nmol·mol^−1^, with an MDL of 0.04–2.8 μg·m^−3^ and an RSD <30% for the relative response factors. While in the studies of Zhou and Feng (2018) and Liang et al. (2021) both used Deans Switch center cutting technology, the FID detector only performed the detection of five C_2_∼C_3_ hydrocarbons, such as ethylene, acetylene, ethane, propylene, and propane, while the other 103 VOCs were still detected by MS. Thus, we used SUMMA canisters to collect VOCs from plastic runway tracks and the collected VOCs were detected and analyzed using GC-FID and GC-MS with dual-pathway injection and dual-pathway detection in this study.

VOCs released from plastic runway tracks can directly jeopardize the health of sports people, but the optimal environmental conditions for the release of VOCs from plastic runway tracks are not clear, and the qualitative and quantitative determination of VOCs from plastic runway tracks is not yet perfect. To elucidate the VOCs released from plastic runway tracks more comprehensively, in this research, the optimal ambient temperature, relative humidity (RH), air exchange rate (AER), and release time for the release of VOCs from the plastic runway tracks were determined. And then, an environmental chamber was adopted to release VOCs from plastic runway tracks. A SUMMA canister was used to collect VOCs in an environmental chamber. The SUMMA canister was connected to the atmospheric pre-concentrator which used to condense VOCs in a three-stage cold trap, and the GC-MS/FID method was used to conduct the qualitative and quantitative analyses of VOCs, and the determination method based on the environmental chamber-canister sampling-three-stage cold trap preconcentration-GC-MS/FID was established. Compared to existing studies on the detection of VOCs on plastic runway tracks, the method developed in this study employs dual-pathway injection and dual-pathway detection of GC-FID and GC-MS, and the VOCs samples are analyzed by MS and FID, respectively, which allows for the simultaneous determination of 101 VOCs released from the plastic runway tracks without compromising the sensitivity. Through this study, the influences of the ambient temperature, RH, AER, and release time on the release of VOCs from plastic runway tracks have been clarified, and the established methodology lays the foundation for the successful and reliable detection and monitoring of VOCs released from plastic runway tracks.

## 2 Materials and methods

### 2.1 Specimen preparation

The investigated PU plastic runway surface consisted of a double-layer structure, i.e., a lower layer and an upper layer, which was commercially obtained from Wuhan Ruitian New Material Technology Co., Ltd. (Wuhan, China) (Liu et al., 2025). The lower layer was composed of PU adhesive and rubber particles, with a rubber content of 15% and a particle size of 2.00–3.00 mm, uniformly mixed at a mass ratio of 3:1. The thickness of the lower layer was 10.00 ± 1.00 mm. The upper layer was composed of PU adhesive and rubber particles, with a rubber content of 20% and a particle size of 1.00–2.00 mm, uniformly mixed at a mass ratio of 1:1.40.00 g of diluent was added to each 2.00 kg PU adhesive. The thickness of the upper layer was 3.00 ± 1.00 mm, while the surface area of the prepared plastic runway track was 300.00 mm × 300.00 mm. It was exposed to outdoor conditions for 20 days, and after fully formed, it was sealed in a polytetrafluoroethylene film and stored at 25°C ± 5°C.

**FIGURE 1:**
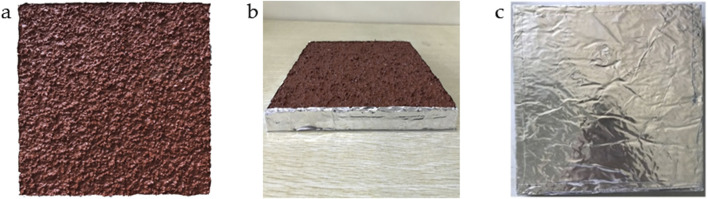
A PU plastic runway track sample after sealing and covering with aluminum foil. **(a)** front, **(b)** side, and **(c)** back surfaces.

The sample was taken out 24 h before the experiment, and a square of 200.00 mm × 200.00 mm was cut 50.00 mm from the sample’s edges. The cut sample was sealed and covered with aluminum foil on the sides and the bottom, ensuring an exposed area of 0.04 m^2^, as shown in Figure 1. Because different plastic runway tracks and different VOCs require different times to reach the release equilibrium (Wu et al., 2019; Yuan et al., 2019), the sample was pre-equilibrated for 24 ± 1 h in an environment free from other sources of VOCs release at an ambient temperature of 23°C ± 2°C and a relative humidity (RH) of 50% ± 10%. Subsequently, the PU plastic runway track sample released VOCs in the environmental chamber.

### 2.2 Instruments and chemicals

The following instruments and chemicals were used in the study: a QP 21-H4L100 Small VOCs release environmental chamber (0.1 m^3^, Shanghai Qinpei, CHN), a gas chromatograph coupled to a mass spectrometer (Agilent 7820A/5977E GC-MS, Agilent, United States), a Gilian IAQ-Pro constant flow air sampling pump (Sensidyne, United States), Silonite SUMMA Canisters (Entech, United States), a 7100A pre-concentrator (Entech, United States), a 7016CA autosampler (Entech, United States), a 3100A canister cleaner (Entech, United States), and a 4600A automatic VOCs dilutor (Entech, United States).

Furthermore, a four-component internal standard mixed standard gas (CH_2_BrCl, C_6_ClD_5_, C_6_H_4_F_2_, C_6_H_4_BrF, 4 ppb, Linde, GER), a PAMS mixed standard gas (C_2_∼C_12_, 57 types, 4 ppb, Linde, GER), a US EPA TO-15 mixed standard gas (65 types, 4 ppb, Linde, GER), deionized water (0.1 μS cm^−1^, HUSHI, CHN), an alkaline cleaning agent (PH ≥ 7.5, HUSHI, CHN), nitrogen gas (purity ≥99.999%, HUSHI, CHN), and helium gas (purity ≥99.999%, HUSHI, CHN) were used.

### 2.3 Experimental process

#### 2.3.1 Instrument pretreatment

The mass concentrations of VOCs in the cleaned VOCs environment chamber and SUMMA canisters were ≤50.00 μg m^−3^, and the mass concentrations of other single pollutants were ≤5.00 μg m^−3^ (Ministry of Education of the People’s Republic of China, 2018). Meanwhile, Silonite SUMMA canisters were evacuated to ≤6.66 Pa when not used (Wei, 2006).

To minimize VOCs interactions with the liner surface, improve trace analyte detection, enhance thermal stability, suppress catalytic reactions, and boost method reproducibility/accuracy, we installed a silanized glass liner at the inlet of the GC-MS instrument (Gerhards et al., 2022; Robbins et al., 2023), and the GC column was aged at 270°C and cleaned with nitrogen as carrier gas. After cleaning, the GC-MS/FID instrument was used for the blank test, and none of the target compounds were detected.

#### 2.3.2 Release of VOCs

Temperature, RH, AER, and other chamber parameters were set. The AER refers to the ratio between the volume of clean air entering the environmental chamber per unit of time and the volume of the test chamber. No-load operation was applied until the parameters in the chamber reached the preset values and stabilized for 1 h. Afterward, the plastic runway track sample was put in the center of the chamber, and the chamber door was closed immediately to release VOCs. The moment when the sample was put into the chamber was recorded as the zero initial time.

#### 2.3.3 Collection of VOCs

After the release of VOCs from the plastic runway track sample for 24 h, the SUMMA canisters were used for the constant current collection of VOCs. The sampling flow was 0.20 L·min^−1^, and the sampling time was 30 min.

#### 2.3.4 Qualitative and quantitative GC-MS/FID analysis of VOCs

The VOCs were fed from the SUMMA canister into the 7100A pre-concentrator using the 7016CA autosampler. The VOCs were pretreated *via* three-stage cold trap concentration steps including glass-bead cold trap concentration, Tenax tube cold trap concentration, and capillary glass tube absorption focusing; then, the VOCs were introduced into the GC-MS instrument for chromatographic separation and mass spectrometry detection.

##### 2.3.4.1 Cold trap concentration conditions

The used coolant was liquid nitrogen. The glass-bead cold trap concentration was performed using a capture time of 5 min at a flow rate of 100.00 mL·min^−1^ at −150°C; the resolution temperature was 10°C, the valve temperature was 100°C, and the bakeout was performed at 150°C for 15 min. The Tenax tube cold trap concentration was conducted at a capture time of 5 min at a flow rate of 10.00 mL·min^−1^ at −15°C; the resolution temperature was 180°C for 3.5 min, and the bakeout was performed at 190°C for 15 min. Finally, for capillary glass tube absorption focusing, the focusing was done at −160°C, at a resolution of 2.5 min, and the bakeout was at 200°C for 5 min. The transmission line temperature was 120°C.

##### 2.3.4.2 Chromatographic measurement conditions

The chromatographic measurements were performed using the following columns:

Column 1: PLOT capillary column, 15 m × 0.32 mm × 3 μm (Agilent, United States).

Column 2: DB-624 capillary column, 60 m × 0.25 mm × 1.4 μm (Agilent, United States).

The PLOT capillary column was connected to the FID detector, and the DB-624 capillary column was connected to the MS detector.

The temperature program was set with an initial temperature of 35°C, which was kept for 3 min. Then, the temperature was increased to 180°C at a heating rate of 6°C·min^−1^ and kept for 7 min. Subsequently, the temperature was increased to 200°C at a heating rate of 10°C·min^−1^ and kept for 4 min. The sample inlet temperature was 200°C. The PLOT column carrier gas (helium, purity ≥99.999%) flow was 1.00 mL·min^−1^, and the DB-624 column carrier gas (helium, purity ≥99.999%) flow was 1.30 mL·min^−1^. The temperature of the FID detector was 200°C, and the solvent delay time was 5.6 min.

##### 2.3.4.3 Mass spectrometry measurement conditions

The interface temperature was 250°C, and electron impact ionization was conducted at 70 eV; the MS quadrupole temperature was 150°C, the auxiliary heating temperature was 200°C, and the ion source temperature was 230°C. The scan range was performed in the range of 35–300 amu.

## 3 Results and discussion

### 3.1 Selection of test conditions for the environmental chamber

#### 3.1.1 Ambient temperature

**FIGURE 2:**
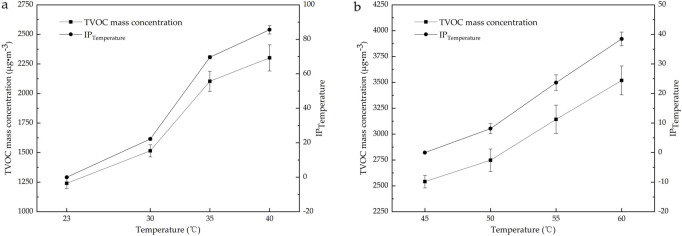
Influence of the ambient temperature on the mass concentration of TVOCs released from the plastic runway track. The average values from n = 3 measurements are shown. **(a)** 23°C–40°C and **(b)** 45°C–60°C.

The changes in ambient temperature alter the thermal motion and vapor pressures of VOC molecules, affecting their adsorption ability but also the capacity of adsorbent materials for the VOC molecules, which is one of the key factors affecting the release of VOCs from materials (An et al., 2007; Wei et al., 2017; Van Der Wal et al., 1991). Based on the pre-equilibrated temperature of 23°C for plastic runway tracks and considering extreme surface temperatures of up to 60°C under prolonged direct sunlight during summer months (Twomey et al., 2014; Gustin et al., 2018), this study investigates the temperature range of 23°C–60°C to evaluate thermal effects. In addition, accounting for both the typical usage environment (≤40°C) and extreme conditions (>40°C) of plastic runway tracks, two representative RH values (20% and 45%) were chosen for each temperature range to represent dry and moderately humid conditions (Liu et al., 2023). As shown in Figure 2, the influence of the ambient temperature, particularly 23, 30, 35, and 40°C at an RH of 45%, and 45, 50, 55, and 60°C at an RH of 20%, on the mass concentration of TVOCs released from the plastic runway track was studied and analyzed at an AER of 1.0 h^−1^ and a release time of 24 h. Figure 2 shows the increase percentage (%) (*IP*_*Temperature*_) in the TVOC mass concentration at high temperatures relative to low temperatures. The calculation formula of the *IP*_*Temperature*_ is as Equation 1:

$$IP_{Temperature}=\frac{TVOC_{x^{{}^{\circ}}C\;}–\;\;TVOC_{23\;or\;45{\;}^{{}^{\circ}}C}}{TVOC_{23\;or\;45{\;}^{{}^{\circ}}C}}\times 100\%$$ (1)

Figure 2 indicates that the concentration of TVOCs from the plastic runway track significantly increases with the ambient temperature. The *IP*_*Temperature*_ increases most between 30°C–35°C. When the temperature exceeds 60°C, it is difficult to reach an equilibrium of the environmental conditions in the environmental chamber, and the time required for reaching stable RH is significantly prolonged. At the same time, the background concentrations of organic contaminants in the chamber are higher, and the pretreatment time of the environmental chamber is longer. To ensure adequate analysis of the release of VOCs from plastic runway tracks, we set the temperature for the release of VOCs from plastic runway tracks in the environmental chamber at 60°C.

#### 3.1.2 Ambient relative humidity

**FIGURE 3:**
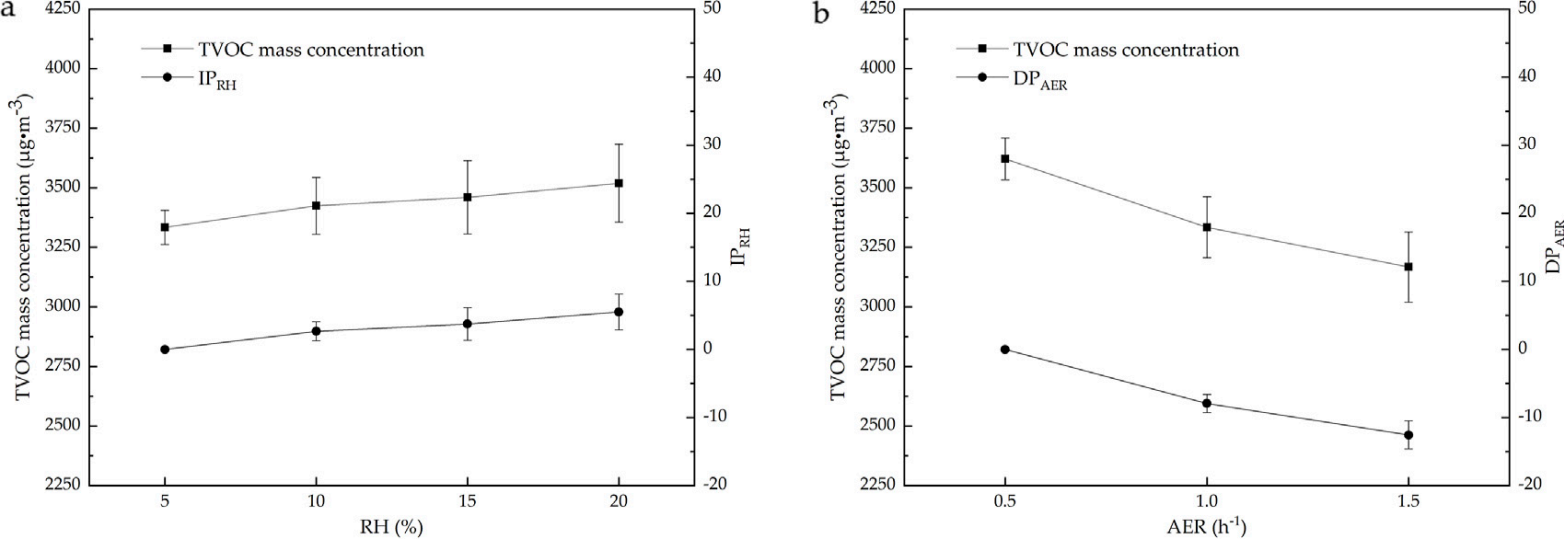
Influence of the ambient RH and AER on the mass concentration of TVOCs released from the plastic runway track. The average values from n = 3 measurements are shown. **(a)** RH and **(b)** AER.

The changes in the ambient RH alter the ambient water vapor pressure and its gradient relative to the internal water vapor pressure of materials. This, in turn, affects the release of VOCs from materials and their adsorption characteristics (Lin et al., 2009; Zhao et al., 2016). According to the ambient temperature of VOCs released from the plastic runway tracks is 60°C and the environmental chamber to achieve the temperature and humidity balance, this study selects the ambient RH of 5%–20% for analysis. As shown in Figure 3a, the influence of the ambient RH, particularly 5, 10, 15, and 20%, on the change in the mass concentration of TVOCs released from the plastic runway track was analyzed at an ambient temperature of 60°C, an AER of 1.0 h^−1^, and a release time of 24 h. Figure 3a also shows the *IP*_*RH*_ in the TVOC mass concentration at high RH relative to low RH. The calculation formula of the *IP*_*RH*_ is as Equation 2:

$$IP_{RH}=\frac{TVOC_{x\%\;}–\;\;TVOC_{5\%}}{TVOC_{5\%}}\times 100\%$$ (2)

Figure 3a indicates that the mass concentration of TVOCs released from the plastic runway track slightly increases with the RH. However, the *IP*_*RH*_ is significantly lower than the *IP*_*Temperature*_, indicating that RH has a significantly lower effect on VOCs released from plastic runway tracks than the temperature. When the ambient temperature is 60°C, higher ambient chamber technology is needed to reach an RH value of 45%, but it also takes longer for the chamber to reach equilibrium environmental conditions. When the RH value is 15%, it takes 8–10 h to reach equilibrium environmental conditions in the environment chamber. When the RH is 20%, the environment in the chamber still greatly fluctuates after reaching equilibrium. This increases the cost of the experiment and significantly reduces its efficiency. Therefore, we studied the VOCs released from plastic runway tracks at an RH of 5% in the chamber.

#### 3.1.3 Ambient air exchange rate

The changes in the ambient AER alter the concentration gradient of VOCs in the boundary layer between the materials and the chamber air flow, influencing the diffusion coefficient and adsorption of VOCs and critically affecting the release of VOCs from materials (Xiong et al., 2013; Manoukian et al., 2016; Deng et al., 2012). As shown in Figure 3b, the influence of the AER, particularly 0.5, 1.0, and 1.5 h^−1^, on the change in the mass concentration of TVOCs released from the plastic runway track analyzed at a temperature of 60°C, an RH of 5%, and a release time of 24 h. Figure 3b also shows the decrease percentage (*DP*_*AER*_) in the TVOC mass concentration from high to low AERs. The calculation formula of the *DP*_*AER*_ is as Equation 3:

$$DP_{AER}=\frac{TVOC_{x\;h^{‐1}}\;–\;\;TVOC_{0.5\;h^{‐1}}}{TVOC_{0.5\;h^{‐1}}}\times 100\%$$ (3)

Figure 3b indicates that the mass concentration of TVOCs released from the plastic runway track decreases significantly with the AER. The higher the AER, the greater the dilution effect of the AER on the released VOCs, failing to detect some trace VOCs. When the AER is very small or zero, a high mass concentration of VOCs results in a significant increase in residual VOCs in the environmental chamber and the GC-MS instrument, directly affecting the background concentration of the environmental chamber and the detection accuracy of the GC-MS instrument. More importantly, plastic runway tracks are usually used outdoors, where the AER value is not zero. Therefore, we set the AER of VOCs released from the plastic track in the environmental chamber as 1.0 h^−1^.

#### 3.1.4 The release time of VOCs

**FIGURE 4:**
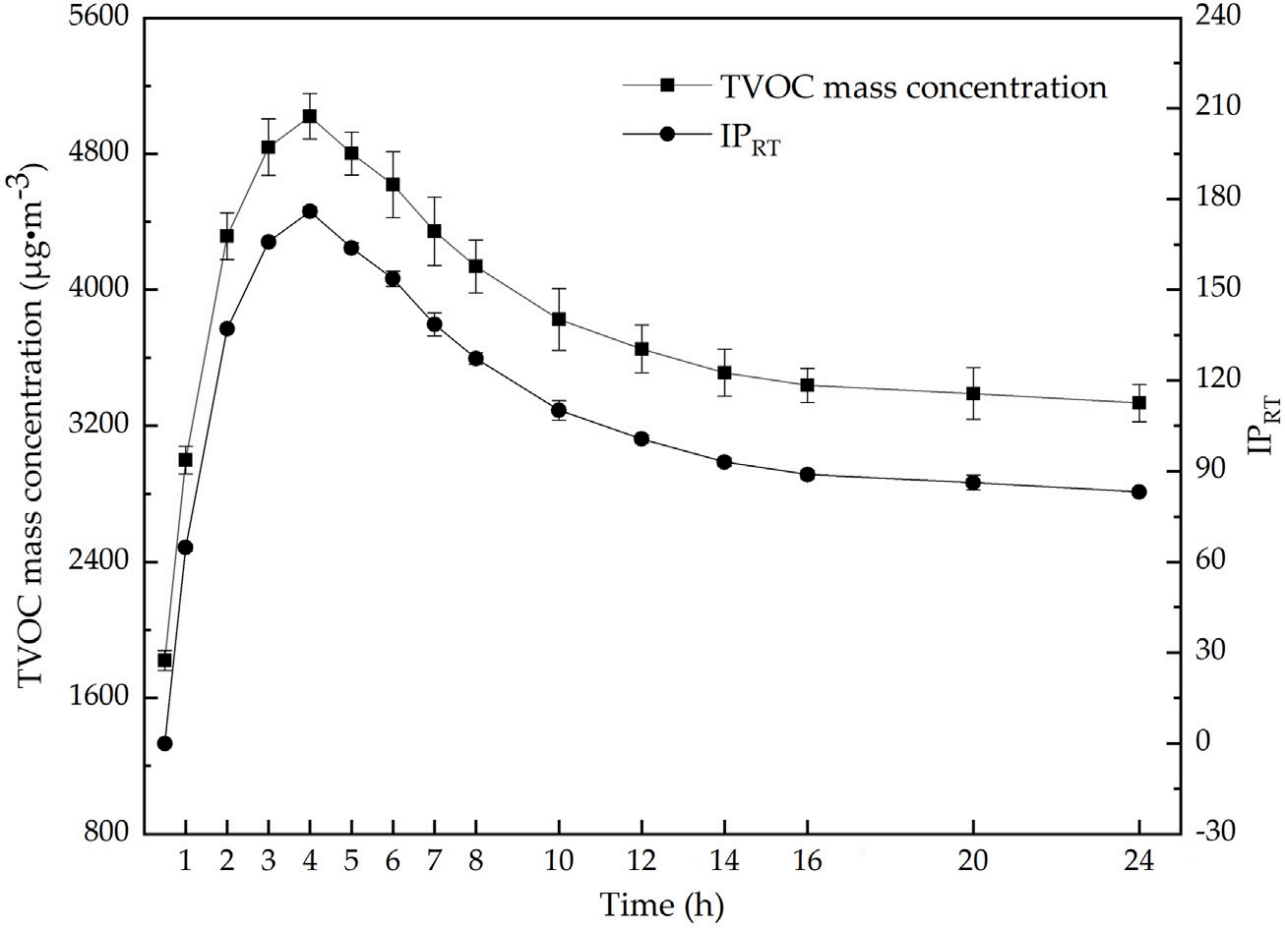
Change in the TVOC mass concentration from the plastic runway track with the release time. The average values from n = 3 measurements are shown.

The release time of VOCs determines whether the investigated material has reached a stable release stage, i.e., whether the mass concentration of VOCs in the environmental chamber tends to equilibrate. When the release time is too short, the release rate of VOCs from plastic tracks is unstable, and there is an inhomogeneous distribution of VOCs in the chamber, which affects the sampling of VOCs. If the release time is too long, the mass concentration of VOCs further decreases, affecting the detection of trace VOCs and reducing the analysis efficiency. As shown in Figure 4, the change rule in the mass concentration of TVOCs released from the plastic runway track with time was studied and analyzed at an ambient temperature of 60°C, an RH of 5%, and an AER of 1.0 h^−1^. Figure 4 also shows the *IP*_*RT*_ in the TVOC mass concentration at each time relative to 0.5 h. The calculation formula of the *IP*_*RT*_ is as Equation 4:

$$IP_{RT}=\frac{TVOC_{x\;h}\;–\;\;TVOC_{0.5\;h}}{TVOC_{0.5\;h}}\times 100\%$$ (4)

Figure 4 indicates that the mass concentration of TVOCs released from the plastic runway track significantly changes at short release times, rapidly rising in the beginning, reaching the maximum value at about 4 h, and then exponentially decreasing. The decreasing trend gradually levels off, eventually reaching an equilibrium at 16–20 h. This is consistent with the research results of Yang et al. (2018), Yuan et al. (2019), and Guo et al. (2020) on the TVOC release rate from plastic runway tracks. Therefore, the release time of VOCs from the plastic runway track in the environmental chamber was set to 24 h in this study.

### 3.2 Precision and accuracy of the GC-MS/FID method

#### 3.2.1 GC-MS/FID dual detection system

**FIGURE 5:**
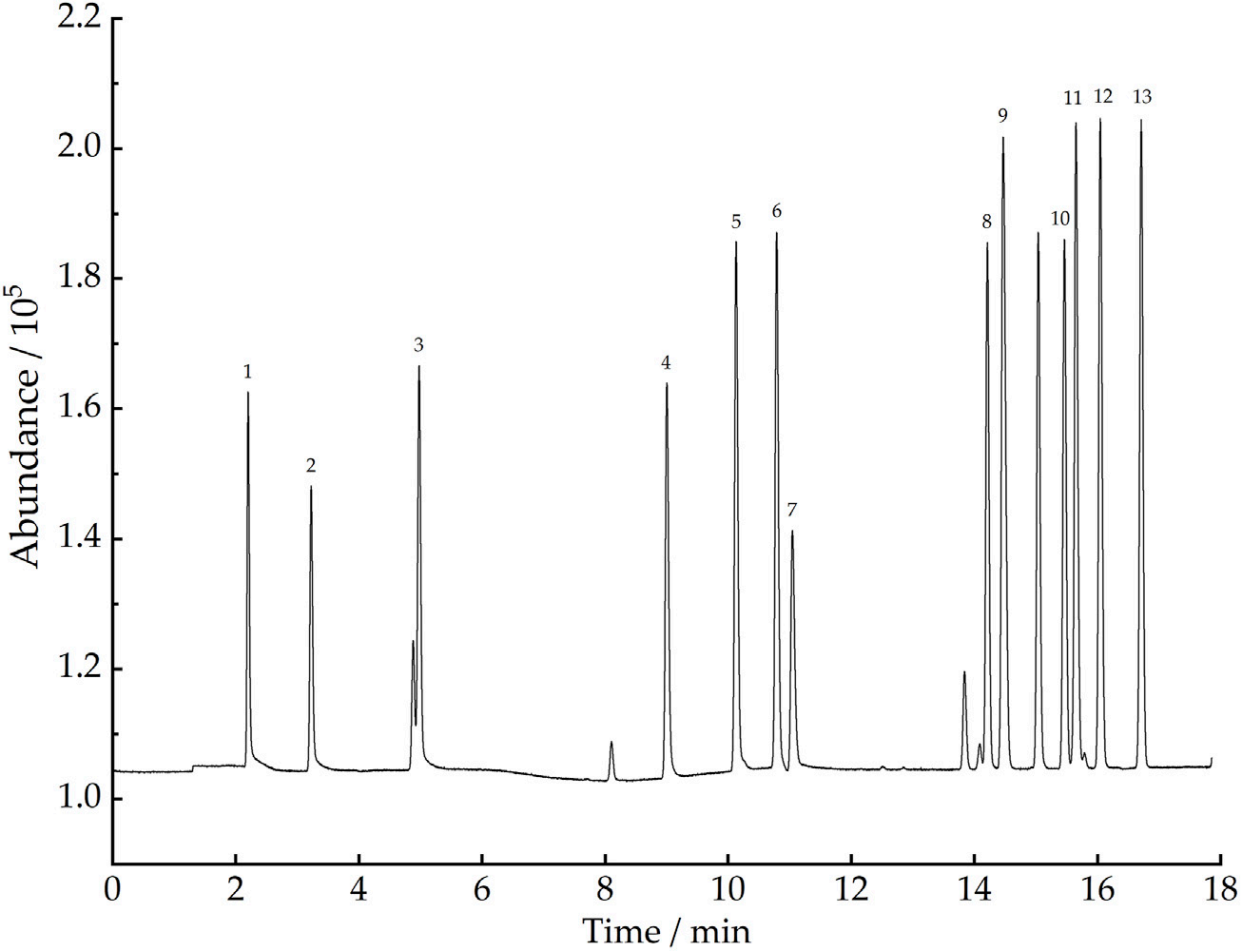
Standard GC-FID chromatogram of 13 (C_2_ - C_5_) hydrocarbon compounds. Compounds: 1) ethane, 2) ethylene, 3) propane, 4) propylene, 5) iso-butane, 6) n-butane, 7) acetylene, 8) trans-2-butene, 9) 1-butene, 10) cyclopentane, 11) cis-2-butene, 12) iso-pentane, and 13) n-pentane.

**FIGURE 6:**
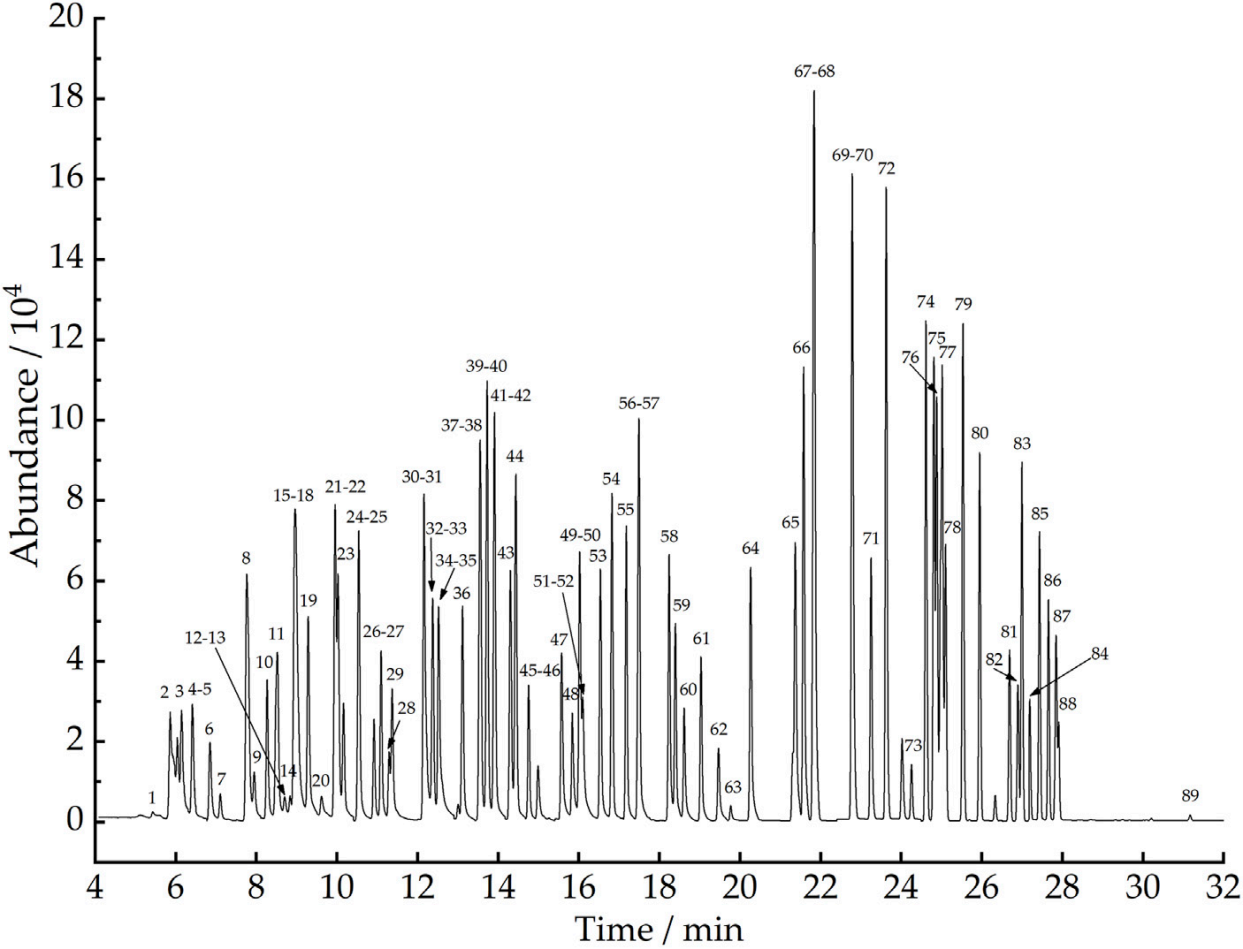
GC-MS total ion chromatogram of 89 compounds. Compounds: 1) dichlorotetrafluoroethane, 2) vinylchloride, 3) chloromethane, 4) 1,3-butadiene, 5) isobutene, 6) bromomethane, 7) chloroethane, 8) trichlorofluoromethane, 9) 1-pentene, 10) trans-2-pentene, 11) isoprene, 12) cis-2-pentene, 13) acrolein, 14) propanal, 15) trichlorotrifluoroethane, 16) 1,1-dichloroethylene, 17) 2,2-dimethylbutane, 18) acetone, 19) methyliodide, 20) acetonitrile, 21) dichloromethane, 22) 2,3-dimethylbutane, 23) 2-methylpentane, 24) tert-butyl methyl ether, 25) 3-methylpentane, 26) n-hexane, 27) 1-hexene, 28) methacrolein, 29) 1,1-dichloroethane, 30) 2,4-dimethylpentane, 31) n-butylaldehyde, 32) methylvinylketone, 33) methylcyclopentane, 34) (Z)-1,2-dichloroethene, 35) methylethylketone, 36) trichloromethane, 37) 2-methylhexane, 38) 1,1,1-trichloroethane, 39) cyclohexane, 40) 2,3-dimethylpentane, 41) 3-methylhexane, 42) carbontetrachloride, 43) sym-dichloroethane, 44) benzene, 45) 2,2,4-trimethylpentane, 46) n-heptane, 47) trichloroethylene, 48) 2-pentanone, 49) methylcyclohexane, 50) 1,2-dichloropropane, 51) n-valeraldehyde, 52) 3-pentanone, 53) bromodichloromethane, 54) 2,3,4-trimethylpentane, 55) 2-methylheptane, 56) trans-1,3-dichloropropene, 57) 3-methylheptane, 58) toluene, 59) n-octane, 60) cis-1,3-dichloropropene, 61) 1,1,2-trichloroethane, 62) tetrachloroethylene, 63) hexanal, 64) 1,2-dibromoethane, 65) chlorobenzene, 66) ethylbenzene, 67) nonane, 68) m-xylene/para-xylene, 69) o-xylene, 70) styrene, 71) bromoform, 72) isopropylbenzene, 73) 1,1,2,2-tetrachloroethane, 74) n-propylbenzene, 75) m-ethyltoluene, 76) p-ethyltoluene, 77) n-decane, 78) 1,3,5-trimethylbenzene, 79) 1-ethyl-2-methylbenzene, 80) 1,2,4-trimethylbenzene, 81) 1,3-dichlorobenzene, 82) 1,4-dichlorobenzene, 83) 1,2,3-trimethylbenzene, 84) benzyl chloride, 85) 1,3-diethylbenzene, 86) 1,4-diethylbenzene, 87) 1,2-dichlorobenzene, 88) n-undecane, and 89) n-dodecane.

This study was conducted to detect 101 VOCs released from plastic runway tracks, the major target of global research on ambient air VOCs that pose different risk factors to human health. The boiling points of the target compounds range from −103.9°C–230°C. The species include 58 C_2_ to C_12_ hydrocarbons, 31 halogenated hydrocarbons, and 13 oxygen-containing aldehydes and ketones. Ethane, ethylene, and other C_2_ to C_5_ low carbon hydrocarbons are more convenient to detect by FID than MS, while other 89 types of target compounds can only be detected simultaneously by MS with a broad-spectrum detector. Therefore, in this study, a GC-MS/FID dual detection system was developed to detect 101 VOCs. After the three-stage cold trap-preconcentrated VOCs entered the GC-MS instrument, the relative retention time of each component in the column was used to determine the type of VOC, the quantitative ion peak area was used to determine the content of each component, and the standard GC-FID chromatogram and the GC-MS total ion chromatogram were plotted. The standard GC-FID chromatogram of 13 C_2_ to C_5_ hydrocarbon compounds is shown in Figure 5, and the GC-MS total ion chromatogram of 89 VOCs, including 31 halogenated hydrocarbons, 45 hydrocarbons, and 13 oxygen-containing aldehydes and ketones, as shown in Figure 6.

In previous studies of VOCs on plastic runway tracks, Hu et al. (2017) determined six benzene series by GC-MS. Yang et al. (2018) used GC with thermal desorption to determine 10 VOCs released from plastic runway tracks. Cui et al. (2019) developed a gas bag sampling-GC-MS for the determination of 18 VOCs. Guo et al. (2020) quantitatively analyzed 35 VOCs using environmental chamber-GC-MS. Li et al. (2020) established a thermal desorption-GC-MS (TD-GC-MS) method for the determination of 10 VOCs. Zhou et al. (2020) determined 16 organic compounds in non-solid raw materials for plastic runway tracks by GC-MS. In the present study, the environmental chamber-canister sampling-three-stage cold trap preconcentration-GC-MS/FID method was established to simultaneously determine 101 VOCs released from plastic runway tracks, with a significant increase in the number of VOC monomers analyzed qualitatively and quantitatively.

#### 3.2.2 Linear relationship of the GC-MS/FID method

Standard gas mixtures were prepared using a 4600A Automatic VOCs dilutor. PAMS mixed standard gas and US EPA TO-15 mixed standard gas were prepared at an initial concentration of 1 ppm and diluted with high-purity nitrogen (purity >99.999%) to 0.8, 1.6, 2.4, 4.0, 8.0, and 16.0 ppb, respectively. A 7016CA autosampler was used to inject the VOC samples at a flow rate of 30 mL/min and an injection time of 10 min for a total of 300 mL. The internal standard method was used for quantification. The linear regression equation and the linear correlation coefficient between each concentration and response value obtained by the least square method are shown in Table 1. According to the established method, the linear correlation coefficients *R* ^*2*^ of 102 VOC monomers are between 0.9546 and 1.0000, and there are 97 VOC monomers with *R* ^*2*^ > 0.9900, including 81 VOC monomers with *R* ^*2*^ ≥ 0.9950.

**TABLE 1:** Linear regression equations, correlation coefficients (*R* ^*2*^), detection limit (in µg·m^−3^ and ppb at 60°C, 1 atm), precision, and repeatability of each VOC monomesr.

No.	Compound name	Linear regression equations	*R* ^*2*^	*MDL* (µg·m^−3^)	MDL (ppb)	*RE* (%)	*RSD* (%)
**1**	ethane	y = 30272x − 945.6	0.9998	0.10	0.091	1.01	1.51
**2**	ethylene	y = 29767x − 24412	0.9973	0.07	0.068	1.10	0.56
**3**	propane	y = 39038x − 38651	0.9945	0.03	0.022	1.11	0.64
**4**	propylene	y = 45191x + 3895.4	0.9999	0.07	0.044	0.35	0.66
**5**	iso-butane	y = 61567x + 1992.6	1.0000	0.10	0.052	1.24	0.36
**6**	n-butane	y = 62643x + 7395.6	1.0000	0.11	0.052	1.09	0.16
**7**	acetylene	y = 31187x + 3667.6	1.0000	0.01	0.010	−0.87	1.58
**8**	trans-2-butene	y = 59211x + 2832.1	1.0000	0.03	0.018	−0.48	0.30
**9**	1-butene	y = 93291x − 41088	0.9833	0.04	0.020	0.42	0.42
**10**	cyclopentane	y = 51486x + 14127	0.9999	0.18	0.090	4.63	0.35
**11**	cis-2-butene	y = 66276x + 12352	0.9999	0.08	0.039	5.24	0.28
**12**	iso-pentane	y = 75259x + 4524	1.0000	0.05	0.024	−0.05	0.52
**13**	n-pentane	y = 75479x + 1556	0.9999	0.06	0.023	0.21	0.55
**14**	dichlorotetrafluoroethane	y = 8263.4x − 17276	0.9546	0.17	0.024	5.13	0.64
**15**	vinylchloride	y = 6948.2x − 4577	0.9969	0.03	0.013	4.95	0.72
**16**	chloromethane	y = 1128.5x − 1029	0.9888	0.04	0.030	2.79	0.98
**17**	1,3-butadiene	y = 320.28x − 236.96	0.9695	0.03	0.015	4.46	1.10
**18**	isobutene	y = 7524.4x − 5459	0.9937	0.03	0.015	5.83	0.79
**19**	bromomethane	y = 9253.9x − 4665	0.9979	0.01	0.005	−0.09	0.70
**20**	chloroethane	y = 2898.6x + 617.64	0.9952	0.07	0.030	2.99	0.53
**21**	trichlorofluoromethane	y = 22533x + 7021.3	0.9968	0.15	0.028	3.36	0.62
**22**	1-pentene	y = 5357x + 872.35	0.9968	0.04	0.016	9.06	0.90
**23**	trans-2-pentene	y = 3251.8x + 218.1	0.9973	0.03	0.012	9.06	0.86
**24**	isoprene	y = 9952.2x − 314	0.9985	0.07	0.028	8.11	1.12
**25**	cis-2-pentene	y = 1494.6x + 197.58	0.9976	0.04	0.016	9.90	0.70
**26**	acrolein	y = 2064.8x + 29.91	0.9974	0.13	0.052	9.83	1.21
**27**	propanal	y = 2789.6x + 53.52	0.9979	0.08	0.036	6.15	0.95
**28**	trichlorotrifluoroethane	y = 18747x + 1025.4	0.9973	0.09	0.012	7.95	0.78
**29**	1,1-dichloroethylene	y = 12682x + 1788.5	0.9973	0.07	0.026	9.30	0.65
**30**	2,2-dimethylbutane	y = 10929x + 1543.4	0.9979	0.06	0.023	8.68	0.97
**31**	acetone	y = 2846.3x + 826.78	0.9986	0.08	0.038	7.93	1.05
**32**	methyliodide	y = 27726x + 4006.2	0.9977	0.03	0.008	2.71	0.83
**33**	acetonitrile	y = 3476.5x + 2056.3	0.9995	0.18	0.088	12.36	2.86
**34**	dichloromethane	y = 6888x + 1315.2	0.9974	0.11	0.046	10.43	0.87
**35**	2,3-dimethylbutane	y = 13162x − 547	0.9976	0.11	0.035	8.81	1.79
**36**	2-methylpentane	y = 13057x + 959	0.9978	0.10	0.032	7.55	2.83
**37**	tert-butyl methyl ether	y = 17558x − 3912.3	0.9986	0.15	0.047	11.12	1.96
**38**	3-methylpentane	y = 16492x + 505	0.9976	0.11	0.035	8.11	1.42
**39**	n-hexane	y = 10872x + 227.8	0.9975	0.10	0.032	8.94	1.52
**40**	1-hexene	y = 7309x + 76.53	0.9976	0.11	0.035	7.90	1.62
**41**	methacrolein	y = 5446x − 497.58	0.9985	0.13	0.051	6.44	1.29
**42**	1,1-dichloroethane	y = 13488x + 432.56	0.9977	0.02	0.009	12.84	0.71
**43**	2,4-dimethylpentane	y = 14988x − 156.59	0.9974	0.13	0.041	7.85	1.49
**44**	n-butylaldehyde	y = 3027.5x − 398.6	0.9981	0.14	0.052	6.88	1.34
**45**	methylvinylketone	y = 3226.7x − 77.6	0.9983	0.14	0.055	9.45	1.46
**46**	methylcyclopentane	y = 14367x + 454.51	0.9977	0.07	0.023	6.83	1.05
**47**	(Z)-1,2-dichloroethene	y = 10144x − 3155	0.9926	0.23	0.086	5.02	0.94
**48**	methylethylketone	y = 3526x − 1465.6	0.9993	0.09	0.035	9.52	1.25
**49**	trichloromethane	y = 18998x − 1.627	0.9975	0.05	0.020	7.81	0.73
**50**	2-methylhexane	y = 14813x − 479.5	0.9978	0.12	0.038	8.08	1.44
**51**	1,1,1-trichloroethane	y = 22092x − 1412.6	0.9982	0.05	0.020	11.11	0.89
**52**	cyclohexane	y = 12453x + 315.6	0.9979	0.04	0.016	8.34	1.03
**53**	2,3-dimethylpentane	y = 10133x + 337.6	0.9975	0.09	0.029	7.68	1.24
**54**	3-methylhexane	y = 11882x + 373.01	0.9974	0.09	0.029	4.87	1.36
**55**	carbontetrachloride	y = 24621x − 439.5	0.9978	0.08	0.017	11.12	0.79
**56**	sym-dichloroethane	y = 11794x − 424	0.9978	0.16	0.066	7.37	0.89
**57**	benzene	y = 26766x − 179.6	0.9976	0.14	0.049	11.62	1.66
**58**	2,2,4-trimethylpentane	y = 6595x − 209.5	0.9975	0.10	0.030	8.49	1.17
**59**	n-heptane	y = 11771x + 39.671	0.9975	0.12	0.038	10.23	1.17
**60**	trichloroethylene	y = 15019x − 2715	0.9979	0.22	0.073	3.32	1.62
**61**	2-pentanone	y = 4039.9x − 5039.5	0.9994	0.35	0.137	−10.16	3.69
**62**	methylcyclohexane	y = 18196x − 906.8	0.9979	0.12	0.039	9.73	1.12
**63**	1,2-dichloropropane	y = 8697.1x − 99.53	0.9972	0.16	0.066	4.83	1.06
**64**	n-valeraldehyde	y = 3682.3x − 2211.6	0.9985	0.22	0.082	7.45	1.48
**65**	3-pentanone	y = 17548x − 14376	0.9984	0.33	0.129	7.95	1.71
**66**	bromodichloromethane	y = 22323x − 2714.5	0.9980	0.08	0.029	8.96	0.75
**67**	2,3,4-trimethylpentane	y = 19836x − 1191.2	0.9977	0.12	0.036	7.64	0.88
**68**	2-methylheptane	y = 14451x − 1329.6	0.9976	0.13	0.041	8.74	0.74
**69**	trans-1,3-dichloropropene	y = 13216x − 5984.3	0.9992	0.18	0.071	6.28	1.50
**70**	3-methylheptane	y = 13056x − 182.6	0.9973	0.11	0.035	5.85	0.70
**71**	toluene	y = 37146x − 3539.6	0.9979	0.46	0.161	4.59	1.28
**72**	n-octane	y = 15924x − 176.8	0.9975	0.24	0.076	8.49	0.75
**73**	cis-1,3-dichloropropene	y = 14922x − 3588.4	0.9980	0.19	0.075	−0.03	1.88
**74**	1,1,2-trichloroethane	y = 12906x − 1387.6	0.9977	0.24	0.100	5.57	1.08
**75**	tetrachloroethylene	y = 12756x − 2443.5	0.9979	0.74	0.154	1.41	1.58
**76**	hexanal	y = 3707x − 5666	0.9959	0.30	0.112	−7.63	3.16
**77**	1,2-dibromoethane	y = 20567x − 4579	0.9979	0.23	0.057	2.45	1.50
**78**	chlorobenzene	y = 30368x − 7097.9	0.9968	0.27	0.089	4.24	1.92
**79**	ethylbenzene	y = 46788x − 7754.6	0.9964	0.38	0.131	8.57	1.55
**80**	nonane	y = 14615x − 3317.56	0.9967	0.61	0.191	7.31	1.40
**81**	m-xylene/para-xylene	y = 65472x − 9912.6	0.9960	0.46	0.158	4.48	1.54
**82**	o-xylene	y = 37226x − 8482.6	0.9969	0.41	0.141	9.02	1.57
**83**	styrene	y = 27381x − 7767.6	0.9967	0.47	0.162	3.94	2.44
**84**	bromoform	y = 27940x − 7657.6	0.9973	0.21	0.046	8.29	1.78
**85**	isopropylbenzene	y = 58571x − 13952	0.9962	0.38	0.131	6.66	1.43
**86**	1,1,2,2-tetrachloroethane	y = 3372.9x − 1356	0.9919	0.49	0.204	7.88	1.85
**87**	n-propylbenzene	y = 54811x − 20492	0.9907	0.51	0.176	6.07	2.04
**88**	m-ethyltoluene	y = 45969x − 18797	0.9908	0.48	0.166	3.88	2.18
**89**	p-ethyltoluene	y = 43669x − 18642	0.9904	0.36	0.124	11.00	2.54
**90**	n-decane	y = 11861x − 3312	0.9929	0.64	0.200	6.61	2.62
**91**	1,3,5-trimethylbenzene	y = 39107x − 16411	0.9907	0.41	0.141	4.74	1.96
**92**	1-ethyl-2-methylbenzene	y = 49531x − 20667	0.9902	0.48	0.166	−0.26	3.06
**93**	1,2,4-trimethylbenzene	y = 35535x − 21583	0.9921	0.46	0.159	−0.44	2.51
**94**	1,3-dichlorobenzene	y = 21566x − 14279	0.9934	0.48	0.199	−5.98	4.94
**95**	1,4-dichlorobenzene	y = 18043x − 12956	0.9942	0.41	0.170	−9.02	3.08
**96**	1,2,3-trimethylbenzene	y = 36844x − 22994	0.9906	0.39	0.135	−8.71	3.00
**97**	benzyl chloride	y = 4045.9x − 5196.7	0.9993	0.20	0.083	−9.79	3.08
**98**	1,3-diethylbenzene	y = 27913x − 26442	0.9933	0.41	0.142	−8.65	2.96
**99**	1,4-diethylbenzene	y = 22336x − 23169	0.9951	0.37	0.128	−6.03	4.34
**100**	1,2-dichlorobenzene	y = 23659x − 16814	0.9927	0.53	0.220	−8.03	3.85
**101**	n-undecane	y = 6909.6x − 9091.7	0.9984	0.53	0.166	−8.18	4.57
**102**	n-dodecane	y = 1351x − 3492	0.9696	0.46	0.144	9.57	4.69

In previous studies, Yuan et al. (2019) used TD-GC-MS to determine VOCs released from plastic runway tracks, and the linear correlation coefficients *R* ^*2*^ were 0.9960 or above. The mean linear correlation coefficients of the TD-GC-MS method established by Kamarulzaman et al. (2019) were 0.9910 ± 0.01, while *R* ^*2*^ > 0.9930 was reported in the gas bag sampling-GC-MS method established by Cui et al. (2019). The environmental chamber-GC-MS constructed by Guo et al. (2020) exhibited *R* ^*2*^ > 0.9900, while Li et al. (2020) developed a TD-GC-MS method with *R* ^*2*^ values >0.9974. In the method established in this study, the linear correlation coefficients for 102 VOC monomers range from 0.9546 to 1.0000, indicating that the established method exhibits good linearity and correlation and is capable of performing qualitative and quantitative analysis of VOCs released from plastic runway tracks.

#### 3.2.3 Detection limit of the GC-MS/FID method

The PAMS mixed standard gas (4 ppb) was mixed with the US EPA TO-15 mixed standard gas (4 ppb), and then the mixed standard gas was diluted to 0.8 ppb using a 4600A automatic VOCs dilutor with high-purity nitrogen (purity >99.999%). The injection flow rate of VOCs was 30 mL/min, the injection time was 10 min, and 300 mL of samples was injected. The measurements were repeated 7 times according to the experimental procedure. The calculation formula of the method detection limit (*MDL*) is as Equation 5:

$$MDL=SD\times t_{\left(n-1,1-\alpha =0.99\right)}$$ (5)

where *SD* is the standard deviation for spiking tests for *n* concentrations in *µg·m* ^−*3*^, *t* is the degree of freedom, and *1- α* is the confidence level.

For the detection limit test of this method, the parallel determination was repeated seven times, and the *t* value is 3.143. Table 1 lists the *MDL* of the method after seven measurements. According to the established method, the *MDL*s of 102 VOC monomers are between 0.01 and 0.74 μg·m^−3^ (equivalent to 0.005–0.220 ppb, at 60°C and 1 atm).

Among the previously established methods used to detect VOCs released from plastic runway tracks, the *MDL* of the environmental chamber-TD-GC method established by Yang et al. (2018) was 0.21–0.41 μg·m^−3^. The *MDL* of the TD-GC-MS method established by Kamarulzaman et al. (2019) was 0.13–3.10 μg·m^−3^. The gas bag sampling-GC-MS of Cui et al. (2019) exhibited an *MDL* value of 0.90–2.20 μg·m^−3^, and for the environmental chamber-GC-MS method of Guo et al. (2020), it was 0.01–0.40 μg·m^−3^. In our method, the *MDL* for 102 VOC monomers was in the range from 0.01 to 0.74 μg·m^−3^, indicating that the established method has a low *MDL* and high sensitivity, which can meet the requirements of the quantitative analysis of low-concentration VOCs in plastic runway tracks.

#### 3.2.4 Precision and repeatability of the GC-MS/FID method

A standard material was adopted for the control experiment. The standard material, with the known concentrations of VOCs, and the actual sample were measured in parallel under the same conditions. The measurement results were compared with the given known value of the standard material to evaluate the accuracy and repeatability of the chosen method.

The mixed standard gas with a concentration of 2.4 ppb was prepared, and the injection time was set to 5 min (the actual injection concentration was 2.4 ppb). The gas was analyzed manually 7 times by GC-MS.

The *RE* was used to calculate the accuracy of the method. The calculation formula is as Equation 6:

$$RE\%=\frac{\bar{x}-c}{c}\times 100\%$$ (6)

where $\bar{x}$ is the average value of seven tests of the sample in *µg·m* ^−*3*^, and *c* is the true value of the standard material in *µg·m* ^−*3*^.

The *RSD* was used to calculate the method’s repeatability. The calculation formula is as Equation 7:

$$RSD\%=\frac{\sqrt{\frac{1}{n-1}\sum_{i=1}^{n}{\left(x_{i}-\bar{x}\right)}^{2}}}{\bar{x}}\times 100\%$$ (7)

where *n* is the number of tests, *x*_*i*_ is the mass concentration of VOC monomers in the *ith* test in *ppb*, $\bar{x}$ is the average mass concentration of VOC monomers in the *nth* test in *ppb*.

After testing, the *RE* and *RSD* determination results of each VOC monomer are listed in Table 1. Table 1 indicates that according to the established method, the *RE* accuracy of 102 VOC monomers ranges from −10.16%–12.84%, while the repeatability *RSDs* range from 0.16% to 4.94%.

In previous studies, the RSD (n = 8) of the environmental chamber-TD-GC method established by Yang et al. (2018) was 1.8%–3.8%. The RSD (n = 6) of the environmental chamber-GC-MS method of Guo et al. (2020) was 1.6%–9.7%. The RSD (n = 6) of the TD-GC-MS method developed by Li et al. (2020) was under 5.0%, the RSD (n = 6) reported by Zhou et al. (2020) for 16 organic compounds in non-solid raw materials for plastic runway tracks by the GC-MS method was below 7.6%. In this study, there are 92 VOC monomers with an RE (n = 7) of less than 10% and only 10 VOC monomers with an RE (n = 7) greater than 10%, with the maximum value of only 12.84%, while the RSD (n = 7) of all VOC monomers is less than 5.00%. It shows that the established method exhibits high consistency, good precision, and excellent repeatability in the repeated analysis of uniform samples.

Given that VOCs released from plastic runway tracks under specific environmental conditions represent instantaneous single-batch samples, and we employed SUMMA canister sampling and completed all analyses within the same day to evaluate short-term accuracy. Consequently, we focused on intraday repeatability (Yuan et al., 2019; Kamarulzaman et al., 2019; Jiang et al., 2019; Cui et al., 2019; Guo et al., 2020; Li H. et al., 2017; Xu, 2017; Zhou and Feng, 2018; Liang et al., 2021). Furthermore, Schanzmann et al. (2025) demonstrated that similar GC-MS methods show excellent interday precision under controlled temperature/humidity conditions, supporting intraday repeatability as a key evaluation metric. We also conducted preliminary interday precision (3 days, n = 3) showing satisfactory consistency (RSD = 1.68%), though interday precision under different environmental conditions warrants additional investigation.

### 3.3 Determination of VOCs released from different plastic runway tracks

**FIGURE 7:**
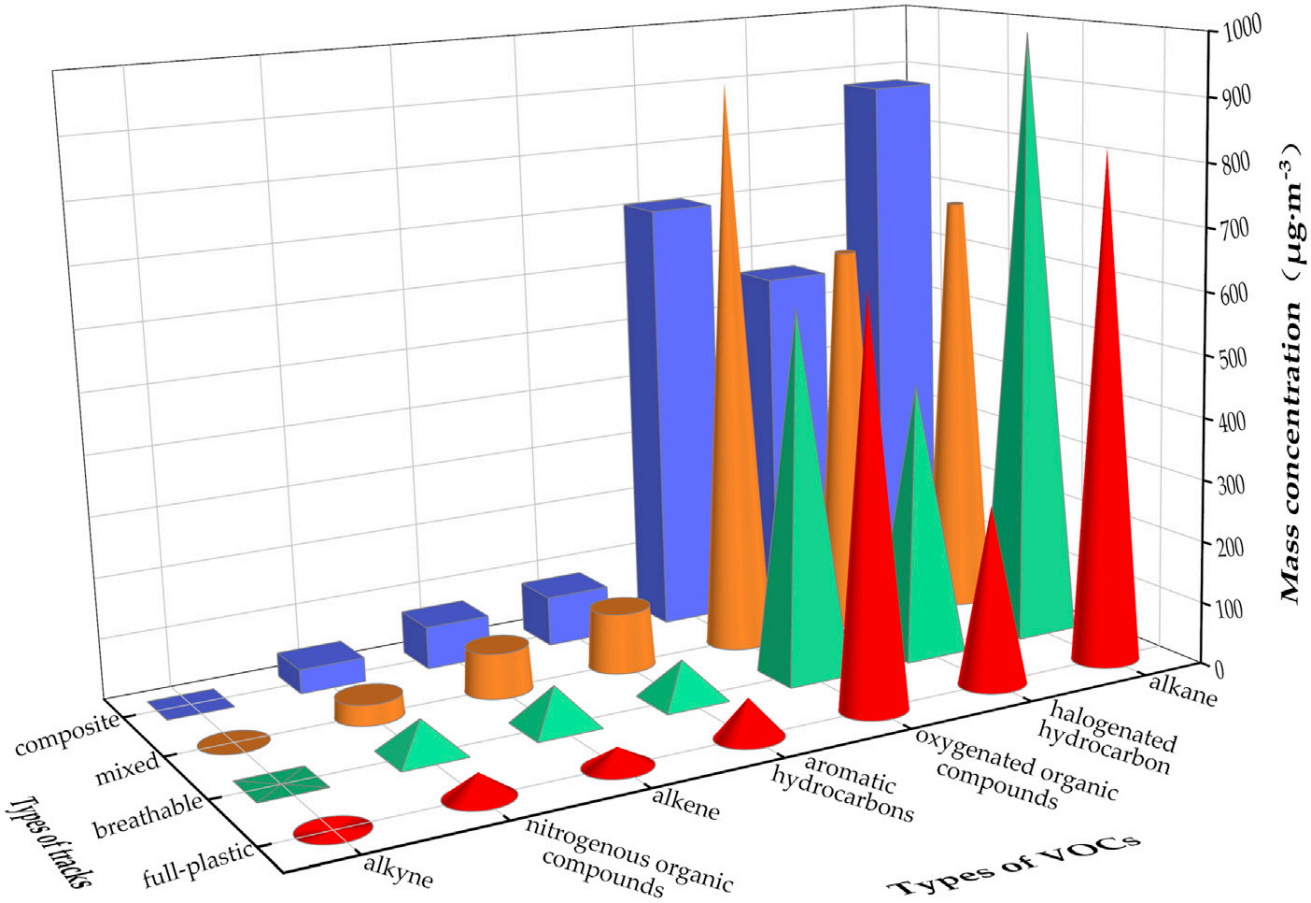
Mass concentrations of various types of VOCs released from four types of plastic runway tracks.

Four types of plastic runway tracks, including composite, mixed, breathable, and full-plastic types, were selected to conduct the qualitative and quantitative analysis of VOCs according to the established determination procedure based on the environmental chamber-canister sampling-three-stage cold trap preconcentration-GC-MS/FID method. When a sample with a large concentration is encountered in the course of sample testing, the sample is diluted a certain number of times using N_2_ and then tested. The results indicate that seven types of VOCs, including alkanes, alkenes, alkynes, aromatic hydrocarbons, halogenated hydrocarbons, oxygenated organic compounds, and nitrogenous organic compounds, are released from the four types of plastic runway tracks at an ambient temperature of 60°C, an RH of 5%, and an AER of 1.0 h^−1^, as shown in Figure 7. The mass concentrations of the various types of VOCs released from the different plastic runway types vary to some extent, but the differences are not significant. Among them, the breathable type released the largest mass concentration of alkanes, followed by the composite type, full-plastic type, and mixed type. The mass concentration of alkenes released from the mixed and composite types runway tracks is larger, and the mass concentration of aromatic hydrocarbons, halogenated hydrocarbons, and oxygenated organic compounds is the largest in the mixed plastic runway tracks, while the mass concentration of nitrogenous organic compounds is the highest in the breathable plastic runway tracks. Overall, alkanes, halogenated hydrocarbons, and oxygenated organic compounds are the main VOCs released from plastic runway tracks.

Regarding the determination of VOC monomers, the mass concentrations released from the four types of plastic runway tracks (Table 2) include aromatic hydrocarbons (e.g., benzene, toluene, ethylbenzene) and halogenated hydrocarbons (e.g., trihalomethanes, carbontetrachloride, methyl iodide, bromomethane, chloromethane), which pose significant hazards to human health. Table 2 shows that the mass concentrations of the 101 VOC monomers released from the four plastic runway tracks also differ to some extent, but the differences are not significant. At the same time, although the detected mass concentrations of acetone, (Z)-1,2-dichloroethene, 3-methylheptane, n-octane, n-decane, n-butane, trans-2-pentene, styrene, 1,1,2,2-tetrachloroethane, and other compounds are relatively high in the four plastic runway tracks, trans-2-pentene, toluene, ethylbenzene, m-xylene/para-xylene, styrene, (Z)-1,2-dichloroethene, acetone, and other compounds are detected in the mixed plastic track with the highest concentration. In the composite plastic runway track, cis-2-butene has the highest detected mass concentration. Propane, iso-butane, 3-methylhexane, 3-methylheptane, n-octane, n-decane, trans-2-butene, chloromethane, dichloromethane, 1,1,2,2-tetrachloroethane, acetonitrile, and other have the highest detected mass concentrations in the breathable plastic runway track, while the detected mass concentrations of n-butane, hexanal, and other compounds are the highest in the full-plastic runway track. The differences in the mass concentrations of 101 VOC monomers in different types of plastic runway tracks may be caused by the differences in the ratio of raw materials and the preparation technology of different plastic tracks.

**TABLE 2:** Mass concentrations of VOC monomers released from the four types of plastic runway tracks.

No.	Category	VOC monomers	Mass concentrations (µg·m^−3^)			
			Composite type	Mixed type	Breathable type	Full plastic type
**1**	**alkanes**	ethane	1.53	1.21	1.07	2.63
**2**		propane	24.47	4.87	35.64	16.39
**3**		iso-butane	15.94	4.15	17.47	16.36
**4**		n-butane	76.75	40.37	97.40	123.31
**5**		cyclopentane	0.32	0.28	0.35	1.35
**6**		iso-pentane	5.52	5.28	4.75	4.77
**7**		n-pentane	2.02	1.44	1.58	1.50
**8**		2,2-dimethylbutane	10.76	8.65	9.88	10.63
**9**		2,3-dimethylbutane	0.11	N.D.	N.D.	0.17
**10**		2-methylpentane	0.87	0.12	0.67	0.66
**11**		3-methylpentane	0.77	0.42	0.67	0.57
**12**		n-hexane	3.03	2.24	3.00	3.40
**13**		2,4-dimethylpentane	2.75	1.60	1.59	1.40
**14**		methylcyclopentane	2.09	1.83	1.93	1.61
**15**		2-methylhexane	0.42	0.14	0.34	0.30
**16**		cyclohexane	1.41	0.51	0.48	0.51
**17**		2,3-dimethylpentane	0.57	1.92	0.30	0.32
**18**		3-methylhexane	13.98	5.19	17.05	9.11
**19**		2,2,4-trimethylpentane	3.47	2.15	3.38	2.95
**20**		n-heptane	3.23	3.04	3.03	2.79
**21**		methylcyclohexane	1.33	1.08	1.32	1.09
**22**		2,3,4-trimethylpentane	2.62	2.01	2.50	3.21
**23**		2-methylheptane	6.45	3.77	6.20	8.27
**24**		3-methylheptane	229.99	185.52	263.50	174.26
**25**		n-octane	247.30	235.93	283.61	227.24
**26**		nonane	4.68	3.78	4.07	4.24
**27**		n-decane	209.11	169.26	222.42	201.30
**28**		n-undecane	3.69	2.90	3.78	3.72
**29**	**alkenes**	ethylene	1.49	1.82	1.43	1.54
**30**		propylene	1.47	1.70	2.72	1.53
**31**		trans-2-butene	0.25	0.14	0.29	0.13
**32**		1-butene	0.41	0.28	0.45	0.45
**33**		cis-2-butene	0.38	0.21	0.26	0.21
**34**		1-pentene	0.04	0.07	N.D.	N.D.
**35**		1,3-butadiene	0.09	N.D.	0.03	N.D.
**36**		isobutene	0.10	0.03	0.15	0.13
**37**		trans-2-pentene	59.08	63.14	52.82	20.88
**38**		isoprene	0.29	0.28	0.27	0.22
**39**		cis-2-pentene	3.48	3.17	2.85	2.84
**40**		1-hexene	3.06	2.14	3.07	2.48
**41**	**alkynes**	acetylene	0.02	0.02	N.D.	0.02
**42**	**aromatic hydrocarbons**	benzene	1.18	1.09	0.99	0.90
**43**		toluene	11.22	13.38	8.19	9.92
**44**		ethylbenzene	9.63	11.86	7.75	6.93
**45**		m-xylene/para-xylene	6.25	8.16	4.38	7.35
**46**		o-xylene	3.35	3.92	3.79	3.51
**47**		styrene	41.33	49.79	30.15	23.63
**48**		isopropylbenzene	1.74	1.94	1.14	2.02
**49**		n-propylbenzene	0.84	0.98	0.88	0.97
**50**		m-ethyltoluene	0.83	0.85	0.59	0.86
**51**		p-ethyltoluene	0.50	0.72	0.37	0.78
**52**		1,3,5-trimethylbenzene	0.43	0.49	0.97	0.53
**53**		1-ethyl-2-methylbenzene	0.49	0.54	0.86	0.48
**54**		1,2,4-trimethylbenzene	1.34	1.85	0.71	2.18
**55**		1,2,3-trimethylbenzene	0.69	0.62	0.64	0.67
**56**		1,3-diethylbenzene	0.68	0.57	1.24	0.65
**57**		1,4-diethylbenzene	1.20	1.08	0.95	1.17
**58**	**halogenated hydrocarbons**	dichlorotetrafluoroethane	2.26	2.31	3.00	1.43
**59**		trichlorofluoromethane	1.31	1.00	1.21	0.94
**60**		trichlorotrifluoroethane	0.13	0.09	0.17	N.D.
**61**		chloromethane	10.77	5.53	18.67	11.97
**62**		bromomethane	0.01	0.02	0.02	0.01
**63**		chloroethane	0.79	0.31	0.80	0.79
**64**		dichloromethane	14.77	8.56	26.27	11.46
**65**		1,1-dichloroethane	N.D.	N.D.	0.08	0.02
**66**		trichloromethane	1.32	1.31	1.35	1.30
**67**		1,1,1-trichloroethane	N.D.	0.06	0.08	N.D.
**68**		carbontetrachloride	5.47	4.77	5.23	2.65
**69**		sym-dichloroethane	2.55	3.12	1.41	1.20
**70**		1,2-dichloropropane	1.28	1.39	1.02	0.95
**71**		bromodichloromethane	0.18	0.14	0.23	0.15
**72**		1,1,2-trichloroethane	0.83	0.41	1.11	1.06
**73**		1,2-dibromoethane	0.31	0.24	0.59	0.25
**74**		bromoform	0.28	0.49	0.63	0.26
**75**		1,1,2,2-tetrachloroethane	83.48	59.73	101.11	86.13
**76**		methyliodide	0.03	N.D.	0.07	N.D.
**77**		vinylchloride	0.13	0.08	0.17	0.12
**78**		1,1-dichloroethylene	N.D.	0.15	0.08	N.D.
**79**		(Z)-1,2-dichloroethene	424.25	528.26	258.41	154.54
**80**		trichloroethylene	0.26	0.29	0.49	0.25
**81**		tetrachloroethylene	3.30	2.69	3.38	2.83
**82**		trans-1,3-dichloropropene	0.20	0.19	0.40	N.D.
**83**		cis-1,3-dichloropropene	0.22	0.41	0.33	N.D.
**84**		chlorobenzene	0.32	0.45	0.61	0.29
**85**		1,3-dichlorobenzene	1.38	1.38	2.67	1.33
**86**		1,4-dichlorobenzene	2.88	1.68	3.39	1.82
**87**		1,2-dichlorobenzene	2.23	1.12	2.49	1.18
**88**		benzyl chloride	5.93	4.50	6.29	4.66
**89**	**oxygenated organic compounds**	acrolein	1.28	1.44	1.04	2.97
**90**		propanal	3.62	6.21	2.97	4.29
**91**		methacrolein	1.71	1.66	1.79	1.32
**92**		n-butylaldehyde	5.68	7.44	4.94	4.68
**93**		n-valeraldehyde	3.71	4.24	3.60	3.24
**94**		hexanal	63.90	70.53	54.75	103.80
**95**		acetone	608.73	823.40	510.25	518.62
**96**		methylvinylketone	1.70	1.64	1.58	1.29
**97**		methylethylketone	11.61	6.13	9.21	7.96
**98**		2-pentanone	3.43	2.18	3.13	2.60
**99**		3-pentanone	1.69	2.45	0.55	1.41
**100**		tert-butyl methyl ether	0.24	0.15	0.20	0.15
**101**	**nitrogenous organic compounds**	acetonitrile	39.70	33.28	55.63	37.82

The detection and analysis of the VOCs in different plastic runway tracks indicate that the established determination method based on environmental chamber-canister sampling-three-stage cold trap preconcentration-GC-MS/FID in this study can be adopted for the qualitative and quantitative determination of 101 VOCs in plastic runway tracks, with strong applicability and good operability.

## 4 Conclusion

The influence of the ambient temperature, RH, AER, and release time on the release of VOCs from plastic runway tracks was systematically analyzed in this study. The optimal environmental parameters for the release of VOCs from plastic runway tracks were an ambient temperature of 60°C, an RH of 5%, an air exchange time of 1.0 h^−1^, and a release time of 24 h. At the same time, the environmental chamber techniques, canister sampling techniques, three-stage cold trap preconcentration techniques, and GC-MS/FID techniques were adopted to collect, concentrate, and conduct qualitative and quantitative analyses of VOCs from plastic runway tracks, respectively. The determination procedure based on the environmental chamber-canister sampling-three-stage cold trap preconcentration-GC-MS/FID method was established, and it could simultaneously determine 101 VOCs released from plastic runway tracks by adopting dual-pathway injection and dual-pathway detection. The 101 VOC monomers determined by the proposed method exhibited linear relationships within a range from 0.8 to 16.0 ppb, with linear correlation coefficients from 0.9546 to 1.0000. The *MDL* range was from 0.01 to 0.74 μg·m^−3^ (equivalent to 0.005–0.220 ppb, at 60°C and 1 atm). The *RE* (n = 7) ranged from −10.16%–12.84%, and the *RSD* (n = 7) varied from 0.16% to 4.94%. The established method is fast, accurate, and sensitive, and thus, suitable for the qualitative and quantitative detection of 101 VOCs in plastic runway tracks. Finally, the quantitative analysis of VOCs released from different plastic runway tracks adopting the established method indicated seven categories of VOCs released from plastic runway tracks, including alkanes, alkenes, alkynes, aromatic hydrocarbons, halogenated hydrocarbons, oxygenated organic compounds, and nitrogenous organic compounds. At the same time, there are some differences in the mass concentration of VOC monomers released from different plastic runway tracks, plausibly originating from different used raw materials and preparation technology.

## Data Availability

The original contributions presented in the study are included in the article/supplementary material, further inquiries can be directed to the corresponding author.
